# Supplementary material for: Multimorbidity and its effect on perceived burden, capacity and the ability to self-manage in a low-income rural primary care population: A qualitative study
Source: PLoS One. 2021 Aug 9;16(8):e0255802. doi: 10.1371/journal.pone.0255802 (PMC8351969; doi:10.1371/journal.pone.0255802)
Supplement: S2 File — (DOCX) [file pone.0255802.s002.docx]

**Additional File 2: Interview Protocol**

**Prior to commencement of interview, review the participant information and consent form, reiterate information and ensure participant understands by asking them to state their understanding of the study.**

A lot of people today have chronic health conditions that require ongoing treatment and self-care. Many people report that looking after their health can become a full-time job in itself. We are interested in finding out how people manage the workload associated with their health and the things that make it harder or easier for them to do this.

First we will complete a survey to find out about your health conditions, the demands they place on your life, and the resources (such as financial and social supports) that you have currently. Then I will be interviewing you to find out more about how you manage your health and what the challenges are for you. I will be recording the interview, so I can remember all that you have said.

We hope that this research will enable us to teach health professionals how to better support people with chronic health conditions and reduce their workload. Everything you say in this session will be confidential and no identifying features (such as your name) will be used in this research. If at any time you want to stop, or have a break, please feel free to let me know.

**Age in years**

**Gender**

**Employment status**

- Employed Full or part time? Occupation:………………………………………..
- On government benefit
  - Age pension
  - Disability pension
  - Newstart (due/not due to health)
  - Single parent pension
  - Other
- Home duties
- Student
- Self-funded retiree
- Other:…………………………………….

**Social status**

Who lives in your house?

- Live alone
- Partner/spouse
- Children Number and ages
- Other (family, friends, housemates) Who and how many:

**Surveys: completed independently by the participant or read aloud as preferred.**

1. Multimorbidity Treatment Burden Questionnaire
2. Disease Burden Impact Scale
3. Adapted Illness Intrusiveness Scale
4. DiP-Care Q

**Interview questions:**

1. **Can you describe to me what your different health problems are?**
2. **Thinking about these different conditions, can you tell me how they affect the rest of your life?**

How do they affect:

- Things at home
  - Household chores
  - Basic personal care such as washing, dressing, eating, sleeping
  - Relationships with family members including any caring responsibilities
  - Other home-based activities like gardening or hobbies
- Things outside the home
  - Socialising with family or friends
  - Hobby leisure or sporting activities
  - Ability to work, study, volunteer.

What are the costs associated with your health conditions?

- Financial costs (appointment fees, medications, other treatments)
- Time costs (travelling, appointments and organising appointments, time to do things like exercise, dietary prep, other treatments etc.)

Of your different health conditions, is there one that is a ‘stand-out’, that you rate as the most important condition? Why this condition?

1. **Next: I would like you to think about the things you do to manage your health problems and the treatments you need to undertake.**

Have you needed to:

- Educate yourself about your health conditions - what they are and how to manage them?
  - How have you learned to do this? From health providers; family/friends; the internet; community support groups?
- Attend appointments?
  - Who, where, how much/often?
- Take medications?
  - How often, how much?
- Monitor your symptoms? (e.g. blood pressure, blood sugar, monitor pain/fatigue levels?)
  - Do you need to do this regularly (how often) or occasionally? Who has taught you or helped you with this?
- Alter your lifestyle – changes in diet, exercise or activity?
  - What do you need to do differently in each of these areas? Who has taught you or helped you with this?
- Deal with your feelings and emotions about your health?
  - How have your health problems affected your mood or changed how you see yourself? What do you do to help you deal with these feelings? Has anyone helped you with this?

Of your different health conditions, is there one that is a ‘stand-out’ in terms of needing more management/having a greater treatment workload? Why this condition?

When thinking about your different health conditions, which areas do you think that you are able to take care of yourself and which areas do you think that health professionals need to do or to help you with?

1. **Now I would like you to think about the difficulties you face when caring for your health.**

Have you had or are you having any difficulty with:

- Educating yourself or finding out about your health problems?
  - Why?
  - Probes if needed: don’t know where to look/what information is safe, health professionals not helpful, literacy/difficulty understanding; mental issues (depression/motivation)
- Attending appointments
  - Why?
  - Probes if needed: time, forgetfulness, transport issues, cost, not sure of benefit, physical issues (pain, fatigue), mental issues (depression/motivation), lack of support?
- Sticking to your medication regime?
  - Why?
  - Probes if needed: forgetfulness, cost, side-effects, worries about benefit; not sure what to do?
- Monitoring your symptoms – e.g. keeping track of symptoms, blood sugar levels etc?
  - Why?
  - Probes if needed: time, forgetfulness, not sure of benefit, physical issues (pain, fatigue), mental issues (depression/motivation), lack of support?
- Following diet/exercise/activity recommendations
  - Why?
  - Probes if needed: time, cost, physical issues (pain, fatigue), mental issues (depression/motivation), worries about benefit, not sure what to do, lack of support?
- Managing your feelings and emotions in relation to your health?
  - Why?
  - Probes if needed: lack of support from family/friends/health professionals, other mental health issues, other life demands making it difficult?

1. **Thinking overall about how you look after your health…:**

- Do you find that sometimes you make a choice between doing the things you need to do to care for your health, and other life priorities?
  - Why? Can you give some examples of this?
- Do you think generally that having more than one health condition makes managing your health more difficult?
  - In what way is it more difficult?
- Is there anything that you think would make it easier for you to take care of your health?
  - How would this thing (or things) help you to manage your health?
  - If you are having difficulty identifying anything, think about the previous things that made it difficult.

1. **Finally I want to ask about the other people involved in your healthcare.**

- Health professionals:
  - Who is involved, what do they do?
  - Have they been helpful or unhelpful? Any difficulties in dealing with them?
  - Are there things that could be improved?
- Family:
  - Who is involved, what do they do?
  - Have they been helpful or unhelpful? Any difficulties in dealing with them?
  - Are there things that could be improved?
- Friends:
  - Who is involved, what do they do?
  - Have they been helpful or unhelpful? Any difficulties in dealing with them?
  - Are there things that could be improved?
- Government services e.g. NDIS, Centrelink, social support services like MFC
  - Who is involved, what do they do?
  - Have they been helpful or unhelpful? Any difficulties in dealing with them?
  - Are there things that could be improved?
- Community services e.g. support groups (local or online), exercise or social groups
  - Who is involved, what do they do?
  - Have they been helpful or unhelpful? Any difficulties in dealing with them?
  - Are there things that could be improved?

1. **End of interview**

Is there anything else you would like to mention about your healthcare that hasn’t been covered? Thank the participant and reiterate that all they have discussed is confidential.
