# Supplementary material for: Multimorbidity and its effect on perceived burden, capacity and the ability to self-manage in a low-income rural primary care population: A qualitative study
Source: PLoS One. 2021 Aug 9;16(8):e0255802. doi: 10.1371/journal.pone.0255802 (PMC8351969; doi:10.1371/journal.pone.0255802)
Supplement: S3 File — (DOCX) [file pone.0255802.s003.docx]

**Additional file 3: Burden and capacity coding**

1. **NPT codes**
2. **Coherence/sense-making work**

**Differentiation:** Initial understanding of situation, risk factors and causes, prioritising treatment and management, instigating behaviour change.

**Communal specification**: learning about the chronic disease (CD) from others – helpful or unhelpful.

**Individual specification:** Learning about the CD yourself (reading, internet etc).

**Internalisation:** Once you have experience of managing the illness: knowing when to seek help, problem solving, developing coping strategies. – or inability to do this.

1. **Cognitive Participation/relationship work**

**Enrolment:** Initial engagement with others for help, adjusting relationships to accommodate new roles due to illness.

**Activation**: Arranging help (treatment, support) from health professionals, social services or friends and family. Difficulties in these areas.

**Initiation:** Organisational activities undertaken as an individual e.g. arranging prescriptions, social care, and transport to appointments or difficulty in doing this.

**Legitimation**: Dealing with difficult relationships (e.g. stigma) with friends, family, HCPs; gaining confidence in correct management.

1. **Enacting Work/collective action**

**Skill-set workability**: Initial routine setting and planning to manage CD

**Contextual integration**: Integrating CD into life: changes to work, finances, social roles caused by CD; difficulties in making these adjustments.

**Interactional workability**: Specific treatment tasks – medications, lifestyle changes, appointments, dealing with side effects. Difficulty in performing those tasks.

**Relational integration:** Maintaining confidence in HCPs, treatment and care plan. Dealing with failures in these areas, e.g. poor care continuity or poor treatment.

1. **Appraisal work/reflexive monitoring**

**Reconfiguration**: Initial alterations to treatment tasks (e.g. medication regimens) to fit with daily activities. Learning new ways of doing things, altering priorities.

**Communal appraisal**: Modifying and appraising management in discussion with others.

**Individual appraisal:** Individual appraisal and modification of treatment approaches.

**Systematisation**: Developing ways of keeping up to date with newly available treatments. Routine self-monitoring.

1. **BREWS codes**
2. **Biography:**

Ability to reframe life and create meaning in life while living with chronic conditions – or inability to do this. Coming to terms with the situation, or difficulty with this. Sense of purpose, meaning, changes in identity.

1. **Resource Mobilisation:**

**Physical:**

- Illness burden: symptoms of pain/fatigue/energy
- Function: effect of CD in terms of daily activities and task performance. General fitness.

**Psychological:**

- Personal - traits such as resilience, self-efficacy, determination; worry, frustration, disorganisation,
- Mental health burden - anxiety, depression
- Cognitive: memory; heath literacy; knowledge of condition; problem-solving/past experience

**Practical:**

- Practical skills or external supports that are available
- Personal: time; transportation; practical skills (e.g. computer literacy) of individual or family
- Financial: income for treatments, loss of income, access to benefits system.
- Organisational: aids, equipment, services supplied by government or NGOs for health or social care

1. **Environment**

**Healthcare:** level of fit between treatment demands and daily life; or poor fit, unsupportive environment, clashing priorities

**Personal:** (e.g. home, workplace, centrelink) good fit as above, or excess demands or inflexible

1. **Work realisation:**

**Treatment workload:** ability to successfully normalise treatment workload; manage competing conditions and deal with healthcare system complexity. Experience of success/failure in this.

**Life demands:** Ability to achieve expected life roles, leisure activities, cultural roles, or difficulty in this.

1. **Social functioning:**

- Ability to socialise with friends, family etc. in a way that is meaningful
- Provision of instrumental/practical support by others
- Ability of the person’s social network to accept their health conditions and adjust relationships successfully/ conversely, stigma and lack of acceptance.
