# Supplementary material for: Multimorbidity and its effect on perceived burden, capacity and the ability to self-manage in a low-income rural primary care population: A qualitative study
Source: PLoS One. 2021 Aug 9;16(8):e0255802. doi: 10.1371/journal.pone.0255802 (PMC8351969; doi:10.1371/journal.pone.0255802)
Supplement: Attachment — Submitted filename: Response to reviewers.docx [file pone.0255802.s004.docx]

**Responses to reviewer comments**

N.B. Line numbers relate to the clean copy of the manuscript, not the marked-up version.

| **Comments: Editor** | **Response** |
| --- | --- |
| Please ensure that your manuscript meets PLOS ONE’s style requirements, including those for file naming. | Headings have been formatted in levels 1,2 and 3. Reference to ‘additional files’ have been replaced by references to files S1, S2, S3 (lines 144, 176, 188). |
| In the Methods section, please provide additional information on the interviewers background and training, the number of interviewers and details regarding the length of the interview and where the interview was conducted. | A sentence has been added in the methods section (lines 168-169) to address this. Specific location and duration details are reported in results, lines 220-221. |
| We noted in your submission details that a portion of your manuscript may have been presented or published elsewhere. “Figure one has been taken from another published manuscript. The figure is available through Creative Commons and the authors have been appropriately cited in the manuscript.” Please clarify whether this publication was peer-reviewed and formally published. If this work was previously peer-reviewed and published, in the cover letter please provide the reason that this work does not constitute dual publication and should be included in the current manuscript. | This publication was peer-reviewed and formally published: Leppin et al, *Healthcare* **2015**, *3*, 50-63; doi:10.3390/healthcare3010050.  Inclusion of this figure does not constitute dual publication since it is a figure for illustrative purposes only. The same figure has been used as an illustration in several other peer-reviewed publications. We also refer to this in the revised cover letter. |
| We note that Figure 1 in your submission contain copyrighted images. All PLOS content is published under the Creative Commons Attribution License (CC BY 4.0), which means that the manuscript, images, and Supporting Information files will be freely available online, and any third party is permitted to access, download, copy, distribute, and use these materials in any way, even commercially, with proper attribution. For more information, see our copyright guidelines: [http://journals.plos.org/plosone/s/licenses-and-copyright](https://protect-au.mimecast.com/s/4rF2Ck8vzZU152LEsJPxr5?domain=journals.plos.org).We require you to either (1) present written permission from the copyright holder to publish these figures specifically under the CC BY 4.0 license, or (2) remove the figures from your submission:4.1.    You may seek permission from the original copyright holder of Figure 1 to publish the content specifically under the CC BY 4.0 license.We recommend that you contact the original copyright holder with the Content Permission Form ([http://journals.plos.org/plosone/s/file?id=7c09/content-permission-form.pdf](https://protect-au.mimecast.com/s/jGY7ClxwA5CpXR9rcY38Wr?domain=journals.plos.org)) and the following text:“I request permission for the open-access journal PLOS ONE to publish XXX under the Creative Commons Attribution License (CCAL) CC BY 4.0 ([http://creativecommons.org/licenses/by/4.0/](https://protect-au.mimecast.com/s/OR6CCmOxBQcDWy0xTNaBbh?domain=creativecommons.org)). Please be aware that this license allows unrestricted use and distribution, even commercially, by third parties. Please reply and provide explicit written permission t4.2.    If you are unable to obtain permission from the original copyright holder to publish these figures under the CC BY 4.0 license or if the copyright holder’s requirements are incompatible with the CC BY 4.0 license, please either i) remove the figure or ii) supply a replacement figure that complies with the CC BY 4.0 license. Please check copyright information on all replacement figures and update the figure caption with source information. If applicable, please specify in the figure caption text when a figure is similar but not identical to the original image and is therefore for illustrative purposes only | Figure 1 has been extracted from the article by Leppin et al listed above. The entire article (including the figure) has been already been published under Creative Commons CC BY 4.0 license and the following statement is included at the end of the article:  © 2015 by the authors; licensee MDPI, Basel, Switzerland. This article is an open access article distributed under the terms and conditions of the Creative Commons Attribution license (http://creativecommons.org/licenses/by/4.0/). |
| We note that you have indicated that data from this study are available upon request. PLOS only allows data to be available upon request if there are legal or ethical restrictions on sharing data publicly. For information on unacceptable data access restrictions, please see [http://journals.plos.org/plosone/s/data-availability#loc-unacceptable-data-access-restrictions](https://protect-au.mimecast.com/s/o4ICCnxyDrCjm9yEf0JcaT?domain=journals.plos.org) | There are legal and ethical constraints on open access to this interview data. This has been imposed by the La Trobe University Human Ethics Committee, on the grounds that it is considered sensitive data that is potentially identifiable. |
| In your revised cover letter, please address the following prompts:a) If there are ethical or legal restrictions on sharing a de-identified data set, please explain them in detail (e.g., data contain potentially identifying or sensitive patient information) and who has imposed them (e.g., an ethics committee). Please also provide contact information for a data access committee, ethics committee, or other institutional body to which data requests may be sent. | See above. This is addressed in our revised cover letter. |
| **Comments: Reviewer 1** | **Response** |
| The theme is relevant and highlights the importance of multimorbidity as an important health indicator, especially in the rural population. However, the article needs to be better reformulated as suggested below | Thank you for your encouraging comments. |
| Introduction: It is long and long-winded and does not address the target audience of the study. There are recent articles, including published in Plos Uma that addresses multimorbidity in a rural population that was not mentioned in this article. I suggest consulting the databases in a systematic way and restructure the introduction, addressing the context of the research problem, the literature gap and the objective / hypothesis you want to achieve. Following is the link to the manuscript that addresses the topic: [https://doi.org/10.1371/journal.pone.0225416](https://protect-au.mimecast.com/s/eIOnCr8DKRUo2QNqI6gfwe?domain=doi.org) | We recognise that the introduction is long and have addressed this. We have also restructured the introduction to address multimorbidity prevalence first (lines 61-65) before moving on to the literature review (with additional references provided) of patient experience, which is the primary focus of this article (lines 75-92). The knowledge gap is referred to in line 123-124 and the aim in line 134-136. We have not specified a hypothesis since this was a qualitative study, designed to generate rather than test hypotheses. |
| Methodology: Would you like the authors to clarify better how the invitation for the research subjects was? How was an approach? Was there any criterion that these items actually portrayed the object of study? Was there any criterion of saturation of the characteristics or was this fact not adopted in the methodology? Describe whether the interview protocol has been previously tested. | The participant invitation process is detailed in lines 158-163. Saturation is reported in line 174. The interview protocol was developed from similar qualitative studies, but not trialled in patients and we have added a sentence stating this in line 168-170. We hope this clarifies these questions and are happy to provide further information if required. |
| Results: The results are very interesting and can be the best result, in the most descriptive and non-analytical style. | Thank you for your comments. |
| Conclusion: too long and repeating results. Highlight the contributions of the study and the social impact of the current scientific production. | We agree with the reviewer that there is some repetition of results and we have addressed this and focussed on the recommendations that have emerged. |
| **Comments: Reviewer 2** | **Response** |
| This study examined multimorbidity and its effect on perceived burden, capacity and the ability to self-manage in a low-income rural primary care population. Even it is a mall quantitative study the results may be useful. However this study has a number of major limitations that make the results difficult to interpret. Therefore, I recommend accepting this paper and publishing it with some major revisions and after the polishing the English language. | Thank you for your comments and we hope we have addressed the concerns expressed. We feel that some of the comments made may relate to a misunderstanding regarding the study design. This is a qualitative study (see title and line 140), not a quantitative study and we apologise if this was unclear. |
| 1. Most studies do not use a hyphen for ‘multimorbidity’. To be consistent with literature, I would suggest to replace ‘multi-morbidity’ into ‘multimorbidity’. | The term ‘multi-morbidity’ is not used in the manuscript and appears only in reference 16. |
| Lines 62-64: the authors mentioned: More recently, there has been a recognition that many chronic conditions do not exist in isolation, but as clusters of conditions. Please add a reference. | Reference added in line 62. |
| Line 64: the authors mentioned: Although prevalence counts vary due to differences…. Please explain, give some examples | This line has been removed due to restructuring the introduction. |
| Line 72: Please give the definition of multimorbidity which was used for your study. | Definition and reference in line 62-3. |
| Lines 88-89: the authors mentioned: The interaction is also subject to a social gradient. This is confusing for the reader. Please provide some explanations. | This section has been rewritten, refer to lines 64-65. |
| Lines 110-112: Authors mentioned that the aim of the study was to explore in a low-income rural population, how the patient experience of multimorbidity affects perceived capacity and workload. Any previous studies which examined that? Any literature for the background for that aim? | Additional references provided both for qualitative studies of multimorbidity lines 79-92 and the use of the cumulative complexity model lines 119-121. Exploration of burden and capacity in this population is new and this is stated in line 123-124. |
| Line 130: the authors mentioned: “We were interested in exploring this in a population with common chronic health conditions...” Please add a reference | This line has been removed in the process of restructuring the introduction. |
| Line 137: I suggest the authors to use just “study design” instead of “overall study design | This has been altered. Line 139. |
| Line 139: authors mentioned that they employed a phenomenological methodology. Please explain. | We have restructured the sentence to provide a definition of phenomenology, line 140-141 |
| Lines 147-148: How did you obtain that information? How did you code and classify which are health condition? Did the authors used specific criteria for disease inclusion? | Health conditions were based on self-report and we have slightly adjusted line 150-151 to reflect this. We used the Disease Burden Impact Scale (line 223) to report and code the specific conditions described in table 3. |
| Line 157: Authors mentioned that participants provided their details with permission. Can you please clarify in the text the approval of the study by a Bioethics Committee as well as if the participants were required to sign a consent form and not why is the case? | We have reported ethics approval in line 142-143 and have added the approval number. In line 166 we have specified completion of written consent. |
| In methods section please provide some details about the measures of the study (variables etc.) | Coding categories for framework analysis are detailed in tables 1 and 2. Since this was a qualitative study, the use of quantitative measures/variables was not relevant. Files S2 and S3 provide further information about interview structure and coding formulation. |
| Line 214: The phone interview due to COVID-19 restrictions need to be mentioned in the limitation section | Addressed in line 574-576 |
| Please clarify why P6, P12 etc. are mentioned between the paragraphs. This is confusing for the reader. It could be mentioned in a table. | P6,12 etc are the participant identification codes, as listed in table 3. We have replaced the ‘P’ with ‘Participant’, to increase clarity in all quotations. |
| Lines 245-248: Split/re-arrange this sentence to make it easier for the reader to read. | This sentence has been reworded Lines 253-255. |
| . Lines 252-253: authors mentioned that loss of physical capacity was strongly associated with perceived conditions importance. It is not specified how the authors find that results. Why strongly? Any test? This is a very crucial aspect for the analyses and thus it needs to be revised. | Since this was a qualitative study statistical tests were not appropriate. We have slightly reformulated the sentence lines 261-264 to make it clearer that this is qualitative data. |
| Line 265: Psychological capacity was also essential. How did the authors find this? Please add details | This was identified by the prevalence of this theme in the coding categories, specifically the intersection of multimorbidity and psychological capacity. The coding categories and description of the analysis process are reported in lines 182-214, tables 1 and 2, figure 2 and file S3. |
| Line 292: Increased healthcare….of income. How did the authors find that association? Is it an association or just an observation? | See above. The association emerged from the coding categories and qualitative analysis process outlined above. |
| Lines 327-328: Please add a reference. | This is a finding that emerged from the data analysed in this study. It does not refer to another study. |
| Lines 355-356: authors mentioned that “this could lead to depression…planning”. Please explain. | This is a finding that emerged from the analysis of the qualitative data. It is further discussed in figure 3, lines 281-287 and 330-333. |
| Lines 441-443: authors mentioned that lifestyle recommendations were often questioned because participants felt they were unrealistic or because people did not know how to undertake exercise when they had coexisting chronic pain. It needs to be mentioned in the limitation section. | For us, this is a relevant qualitative finding that we address in some detail in the discussion lines 524-529. We would not consider this to be a limitation. |
| Line 480: Multimorbidity was associated with significantly more treatment. Why significantly? Any test? This is a very crucial aspect for the analyses and thus it needs to be revised | We have replaced the term ‘significantly’ to make it clearer that this is qualitative data. Line 487. |
| In Discussion section no comparison has been made with other studies. Why were those comparisons not included in the discussion? | Although we do not have a specific paragraph solely focussed on comparison with other studies, we have incorporated relevant studies throughout our discussion. See lines 488, 494, 501, 523, 529, 545, 567 and see also final comment by reviewer 3. |
| **Comments: Reviewer 3** | **Response** |
| **Reviewer #3**: 1. Abstract – clear and summarises the study appropriately | Thank you for your comment. |
| 2. Introduction – sets the context for the study. Provides rationale for undertaking the work. Conveys nuance in understanding and treating multimorbidity | Thank you for your observation. In response to your comments and the other reviewers we have restructured the introduction. |
| Line 93-106 - It was not clear to me how ‘workload’ within the CCM applies to the burden of multimorbidity – please clarify for the naïve reader. | This has been clarified in lines 107-109. |
| It was not clear how the argument that disadvantaged populations are at increase risk of capacity-workload imbalance was reached. Please provide evidence for this assertion. | This has been clarified in lines 109-115. |
| Line 110 - Explain further how ‘multimorbidity is an encounter with complexity’ and for *whom.* | This has been removed in the process of restructuring the introduction. |
| Line 139 - You say you employed phenomenology – how so? Expand and revisit its utility in the discussion briefly. | This has been revisited in line 580-582. |
| Participant recruitment - You say the study focusses on low income rural primary care patients but your participant recruitment description does not make it clear that this was the population you recruited. Nor is this necessarily clear in the participant table, even if the ‘source of income’ suggests low income. | We have added footnotes to table 3 to provide specific income information (lines 236-237). We have slightly reworded lines 148-149 and added a reference to make it clear that community health services in this setting are designed specifically for low income populations. |
| The findings are well-presented and it is clear how the framework analysis has been applied. | Thank you for your comments. |
| . Line 140 – this phrasing suggests quantitative analyses were conducted – rephrase. | We think this may refer to the wrong line number. If the reviewer was referring to line 480, we have replaced the term ’significantly’ to avoid confusion. |
| Line 110-128 I was asking the question where this study aims to rework Shippee’s orginal model and, if so, should this be done at the discussion stage based on data-driven induction? (e.g. application of Theory of Patient Capacity, NPT). Though I have noted you used framework analysis. Moving then onto the discussion, I would suggest you make your enhancement/reframing of the model in line with findings explicit in the discussion. | We have discussed this further in lines 506-515. |
| The discussion roots the findings in pre-existing literature. | Thank you for your comments. |
